# Acute Kidney Injury and Viral Myocarditis Secondary to Acute Epstein-Barr Virus Infection

**DOI:** 10.7759/cureus.83061

**Published:** 2025-04-27

**Authors:** Catarina Santos Reis, Bernardo Fernandes, Pedro Lisboa Gonçalves, Ricardo Neto, Roberto Silva, Isabel Camões

**Affiliations:** 1 Internal Medicine Department, Unidade Local de Saúde São João, Porto, PRT; 2 Nephrology Department, Unidade Local de Saúde São João, Porto, PRT; 3 Medicine Department, RISE-Health, Faculty of Medicine, University of Porto, Porto, PRT; 4 Pathology Department, Unidade Local de Saúde São João, Porto, PRT; 5 Anatomic Pathology Department, Unidade Local de Saúde São João, Porto, PRT

**Keywords:** epstein-barr virus, infectious mononucleosis, myocarditis, renal dysfunction, viral tonsillitis

## Abstract

Epstein-Barr virus (EBV) typically causes a self-limiting disease characterized by fever, pharyngitis, and lymphadenopathy. Clinically overt renal and cardiac involvement is rare, and its presentation ranges from subclinical manifestations to severe organ dysfunction. In cases where renal biopsies have been performed, the most common pathology identified is interstitial nephritis.

We report a case of a 24-year-old male with a history of recurrent tonsillitis - the most recent episode occurring one month prior - who presented to the Emergency Department with a three-day symptom of asthenia, diffuse abdominal pain, and vomiting. His blood tests showed acute kidney injury (AKI) and elevated natriuretic peptide levels, with negative troponin levels. Viral markers were consistent with primary EBV infection. Renal biopsy revealed moderate acute tubular necrosis, with no glomerular injury. Renal function showed signs of improvement within the initial three days following the initiation of fluid therapy. Given the high index of clinical suspicion, a cardiac MRI was performed, revealing myocarditis. This case underscores the potential for EBV to present with AKI and myocarditis, contributing valuable insights into the diverse clinical manifestations of EBV infection.

## Introduction

Epstein-Barr virus (EBV) is a DNA virus belonging to the Herpesviridae family, with a high global prevalence, infecting approximately 90% to 95% of adults worldwide and reaching nearly 100% seroprevalence in developing countries. Transmission occurs primarily through intimate contact with saliva, most notably via kissing, but can also happen through respiratory droplets, sharing of utensils, blood transfusion, and, rarely, sexual contact. In populations with lower socioeconomic status, primary infection typically occurs in early childhood and is often asymptomatic or presents with mild upper respiratory symptoms. In contrast, in developed countries, the peak incidence of infectious mononucleosis (IM) is observed among adolescents and young adults, particularly those aged 15 to 24 years. In these cases, infection classically manifests as IM, characterized by fever, acute pharyngitis or tonsillitis, cervical lymphadenopathy, and atypical lymphocytosis [1-3]. The clinical course is generally self-limited, although severe complications may occur, including renal and cardiovascular involvement [4-8].

The etiological spectrum of viral myocarditis is broad and not limited to EBV. Other well-recognized viral agents include Coxsackievirus B (enterovirus), adenovirus, parvovirus B19, human herpesvirus 6, cytomegalovirus, and influenza virus, as well as others such as HIV and SARS-CoV-2. These viruses can induce myocardial inflammation through various pathophysiological mechanisms, including direct invasion of myocardial cells, viral cytotoxicity, activation of innate and adaptive immune responses, and immune-mediated injury involving autoantibody production and pro-inflammatory cytokines. This process ultimately results in necrosis, dysfunction, and potentially fibrosis of cardiac tissue. Understanding these diverse etiologies and mechanisms is essential for the accurate diagnosis and effective management of cardiac complications associated with viral infections [1-3].

## Case presentation

A 24-year-old Caucasian male with a history of recurrent tonsillitis - six episodes in the preceding year, the last one occurring one week prior - presented to the Emergency Department with marked asthenia, diffuse abdominal pain, and watery vomiting over three days. On examination, he was afebrile (36.8ºC), normotensive (BP 102/64 mmHg), and tachycardic (HR ~100 bpm). Physical examination revealed bilateral tonsillar hypertrophy and hyperemia without exudates, an innocent abdomen, and no mucocutaneous lesions, lymphadenopathy, or organomegaly. His blood tests showed normocytic normochromic anemia (Hb 12.8 g/dL), leukocytosis (4,800 cells/µL), and normal platelet count. Liver transaminases were normal, with a slightly elevated total bilirubin of 1.7 mg/dL due to the indirect fraction. The electrolyte panel showed no abnormalities, but creatinine was elevated to 2.5 mg/dL and blood urea nitrogen was 98 mg/dL, indicating impaired renal function with preserved urine output. Brain natriuretic peptide (BNP) levels were 1,200 pg/mL, and high-sensitive troponin I was < 1.9 ng/dL. C-reactive protein was negative. The analytical results are shown in Table 1. Previous baseline values were unavailable for comparison. Electrocardiogram showed sinus tachycardia (110 bpm) with nonspecific repolarization changes and no ST abnormalities. Chest x-ray was unremarkable. Urinalysis showed minimal findings. A 24-hour urine collection revealed proteinuria of 400 mg with sterile culture.

**Table 1:** Patient's analytical results and reference values. BNP: Brain natriuretic peptide

Parameter	Patient value	Reference range
Hemoglobin	12.8 g/dL	13.5–17.5 g/dL
White blood cells	4,800 cells/µL	4,000–10,000 cells/µL
Platelet count	174,000 cells/µL	150,000–450,000 cells/µL
Total bilirubin	1.7 mg/dL	0.3–1.2 mg/dL
Alanine aminotransferase	32 U/L	7–56 U/L
Aspartate aminotransferase	28 U/L	10–40 U/L
Gamma-glutamyl transferase	35 U/L	9–48 U/L
Alkaline phosphatase	95 U/L	44–147 U/L
Creatinine	2.5 mg/dL	0.6–1.2 mg/dL
Blood urea nitrogen	98 mg/dL	7–20 mg/dL
Sodium	139 mEq/L	135–145 mEq/L
Potassium	4.2 mEq/L	3.5–5.0 mEq/L
BNP	1,200 pg/mL	< 100 pg/mL
High-sensitivity troponin I	< 1.9 ng/mL	< 1.9 ng/mL
C-reactive protein	0.4 mg/dL	< 0.5 mg/dL

Abdominal ultrasound showed normal-sized kidneys and small-volume peri-hepatic ascites, with no hepatosplenomegaly. Autoimmune tests, including serum antinuclear antibodies, antineutrophil cytoplasmic antibodies, complement system, antiphospholipid antibodies, and serum and urine protein electrophoresis with immunofixation, were altogether normal. Infectious screening was negative for human immunodeficiency virus. Cytomegalovirus IgG was positive, with absent IgM titers. Serologies for hepatitis B and C, syphilis, and Parvovirus B19 were all negative. Serum albumin was 36.4 g/L, and total cholesterol was 185 mg/dL, and antistreptolysin O titer was within normal limits. EBV protein creatinine ratio (PCR) and IgM antibodies both positive, confirming acute infection.

**Figure 1:**
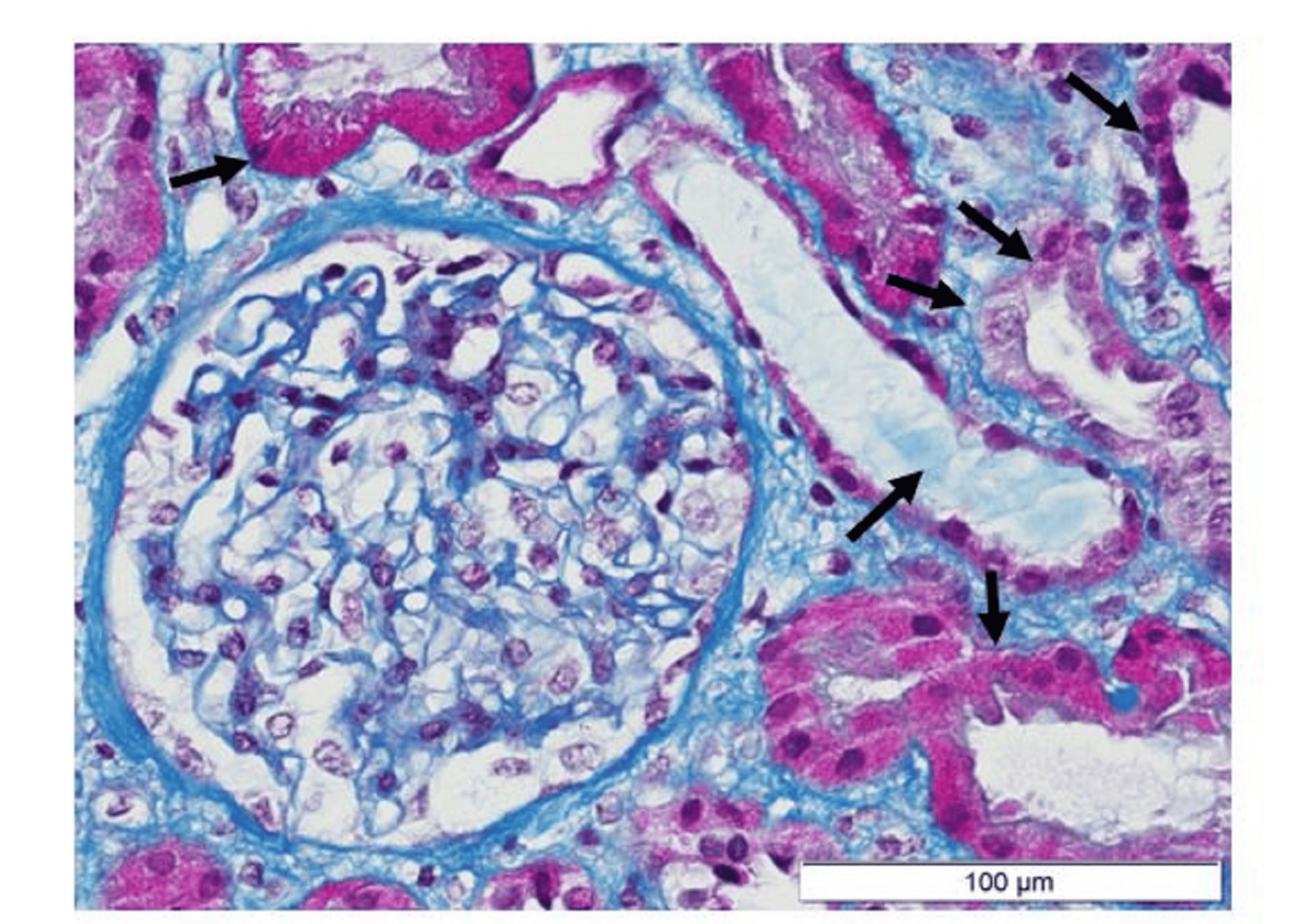
Kidney biopsy with acute tubular necrosis with no other relevant findings (Masson’s trichrome stain – 400x). Arrows indicate areas of tubular injury and tubular casts.

**Figure 2:**
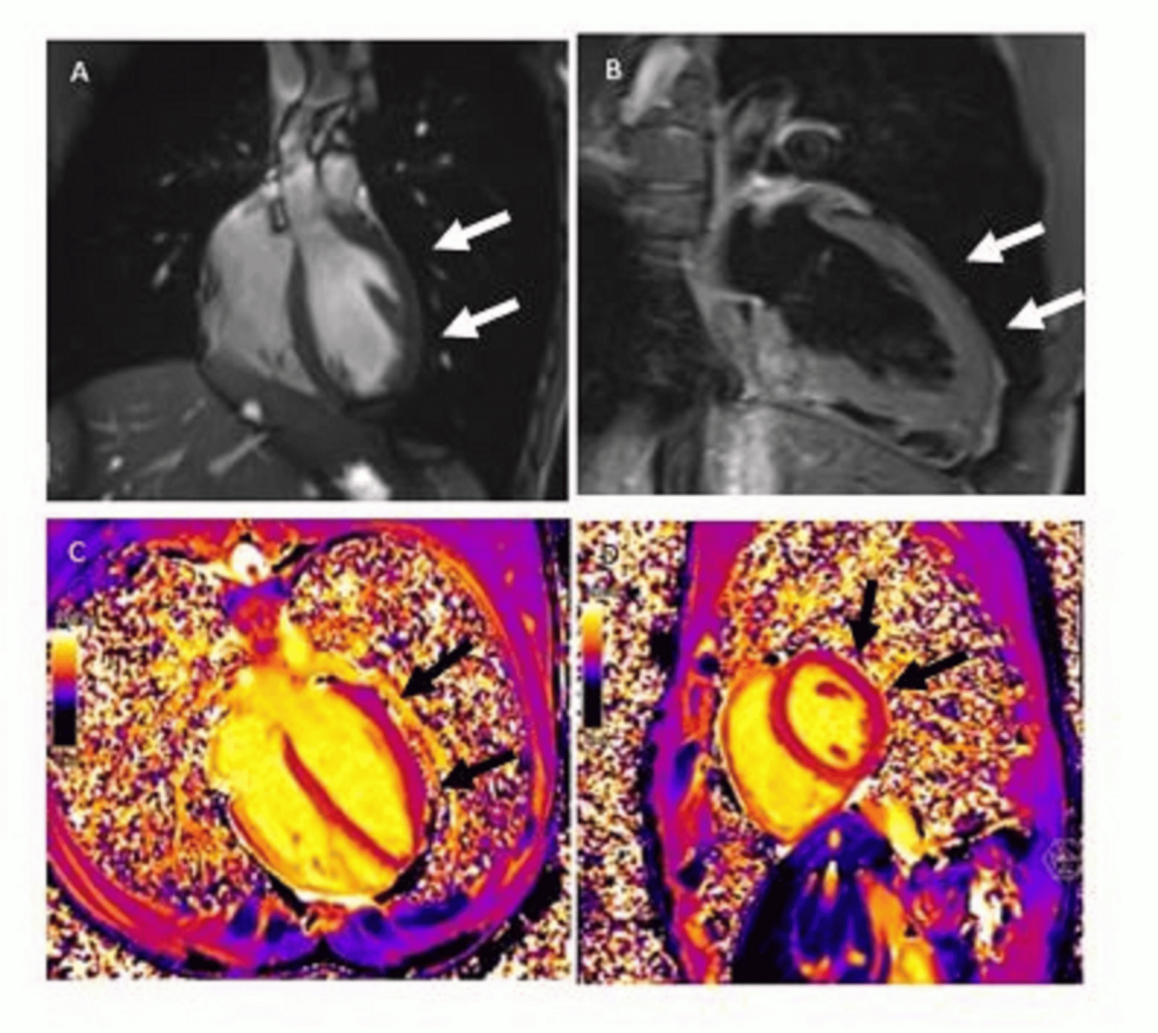
Cardiac magnetic resonance T2-weighted (A, B) and T2-mapping (C, D) images showing myocardial edema in the infero-septal mid-segment.

Persistent renal dysfunction despite support therapy led to renal biopsy performed on the sixth day of hospitalization, which revealed moderate acute tubular necrosis and no glomerular injury (Figure 1). Due to ongoing dyspnea, a transthoracic echocardiogram (TTE) was performed, showing no significant abnormalities. In this context, and given the high index of clinical suspicion, a cardiac MRI was conducted, documenting an edematous area in the infero-septal mid-segment suggestive of myocarditis (Figures 2A-2D). The patient’s condition improved solely with supportive care, and by the eighth day of hospitalization, his creatinine had dropped to 1.5 mg/dL. He was discharged on a RAAS blockade (lisinopril), initiated for cardiorenal protection, and physical exercise was advised to be limited for the following three months. Follow-up EBV serology at day 28 revealed positive IgG Epstein-Barr nuclear antigen (EBNA) titers. Repeated TTE showed no abnormalities one month later. Subsequent follow-up over the next few months showed urine PCR (UPCR) <500 mg/g, and serum creatinine normalized to 0.8 g/dL. Currently, the patient remains asymptomatic and under conservative management with regular outpatient follow-up.

## Discussion

EBV infection often goes undetected if the typical triad-fever, lymphadenopathy, and pharyngitis-is absent. Although most cases resolve spontaneously, severe complications involving the kidney and cardiovascular system may occur as a result of both direct viral damage and immune-mediated mechanisms. While most patients recover without sequelae, several acute complications are associated with this involvement of EBV infection [4-8].

EBV has been linked to various renal syndromes, though the pathophysiological mechanisms are not yet fully understood. Renal involvement has been reported in 3%-16% of patients with acute IM [5,6]. Subclinical renal involvement is common, but associations with parenchymal kidney diseases are rarely reported. One study found an incidence of 11% hematuria and 14% proteinuria in these patients [7]. Renal biopsies in patients without clinical evidence of kidney disease have revealed glomerular edema and localized interstitial inflammation. The most commonly described renal lesion is acute tubulointerstitial nephritis [6]. EBV DNA has been detected in renal tissue in conditions like IgA nephropathy, injury, fibrinogen deposits, immunoglobulin deposits, membranous nephropathy, and focal/segmental lesions, suggesting a causative role [8]. Acute renal failure due to EBV is rare, with a 1996 review identifying 27 cases over 30 years. Of 18 patients, 13 underwent renal biopsy, with 10 showing interstitial nephritis [9]. Laboratory findings and clinical course in our patient were similar to those previously reported, although many of those patients were not subjected to renal biopsy due to earlier renal function improvement. In a few reports, recovery occurred without any targeted treatment, as in our patient. Prognosis is generally favorable, although some cases published report mortality as an outcome, and one patient required a renal transplant [9].

EBV infection affecting the heart and developing myocarditis is a rare event in immunocompetent hosts. Seven cases of EBV-related myocarditis have been reported in the literature, with the first identified by authors in Portugal [4,10]. In most cases, gastrointestinal or respiratory symptoms herald myocarditis. It is typically described as affecting young adult males. The absence of pathognomonic findings and the broad spectrum of clinical manifestations - ranging from subclinical cases to malignant arrhythmias and/or severe systolic dysfunction - make diagnosis challenging [10,11]. In young individuals, significant cardiac injury is uncommon [10,12]. Transthoracic echocardiography (TTE) is the first-line imaging method when myocarditis is suspected, but its accuracy is limited due to the lack of specific echocardiographic findings. Definitive diagnosis of myocarditis is made via endomyocardial biopsy, though it may not always be reasonable, particularly in stable patients with presumed etiology. The European Society of Cardiology diagnostic criteria include at least one clinical feature between acute chest pain, dyspnea or fatigue with onset or worsening, palpitations, arrhythmia, or cardiogenic shock, plus at least one diagnostic finding between ECG changes, elevated troponin, functional or structural cardiac abnormalities on imaging, or characterization of myocardial tissue via MRI [11,13,14]. Cardiac MRI is a useful, non-invasive tool to diagnose myocarditis, detect subclinical cases, and monitor disease progression - hence, it is the reference non-invasive technique. It has a high capacity for detecting interstitial edema during acute inflammation [11]. In our case, the diagnosis was made based on fatigue and MRI findings compatible with myocarditis. Endomyocardial biopsy was not performed due to the use of non-invasive tools and clinical presentation. To date, there is no specific treatment based on available evidence for virus-induced myocarditis [10-12]. The prognosis of myocarditis depends on multiple factors, primarily the severity at presentation, which has prognostic value, and the location of the disease. It generally has a worse prognosis if it progresses to dilated cardiomyopathy, malignant arrhythmias, or heart failure. Although all reported cases resulted in mortality, cardiac causes were confirmed in only one patient [4]. Our patient recovered from the illness with no cardiac or other organ failure and remains asymptomatic.

## Conclusions

This case illustrates an uncommon yet clinically significant presentation of EBV infection with concurrent renal dysfunction and myocarditis in a young, otherwise healthy patient. This case emphasizes the need for a high index of clinical suspicion and highlights the importance of including EBV infection complications in the differential diagnosis of renal and/or cardiac dysfunction in patients with unknown diagnoses. Additionally, in contrast to the other previously published cases, our report presents a survivor of this illness. In the appropriate clinical context, routine testing for EBV serology in patients with unexplained renal and/or cardiac dysfunction may reveal a higher prevalence of EBV-related complications than previously recognized.
